# New 2030 Global Targets for Histoplasmosis from International Society for Human and Animal Mycology (ISHAM) 2025 Histoplasmosis Working Group

**DOI:** 10.3201/eid3203.251165

**Published:** 2026-03

**Authors:** Alessandro C. Pasqualotto, David W. Denning, Thuy Le, Nelesh P. Govender, Ferry Hagen, Rosely M. Zancope-Oliveira, Diego H. Caceres, Ugo Francoise, Allan Guimaraes, Lisandra S. Damasceno, Diego R. Falci, Beatriz L. Gomez, Ilan Schwartz, Jose E. Vidal, Luis E. Galan, Melissa O. Xavier, Mathieu Nacher, Guillermo G. Effron, Gordon Brown, Nicolas Barros, Cassia M. Godoy, Taiguara Fraga, Renata B.A. Soares, Cecilia B. Severo, Alexandre V. Schwarzbold, Indira Berrio, Marineide G. de Melo, Nicole Reis, Bernardo G. Tenorio, Terezinha M.J. Leitao, Claudilson J. de C. Bastos, Monica B. Bay, Marcus V.G. de Lacerda, Luana C.G. Bazana, Daiane F. Dalla Lana, Tarsila Vieceli, Cezar V.W. Riche, Eduardo Arathoon, Cristina Canteros, David Boulware, Ana Alastruey-Izquierdo, Rita Oladele, Marcus de M. Teixeira, Arnaldo L. Colombo, Freddy M. Perez, Tom Chiller, Nathan C. Bahr, Juan L.R. Tudela, Antoine Adenis

**Affiliations:** Federal University of Health Sciences of Porto Alegre, Porto Alegre, Brazil (A.C. Pasqualotto, C.B. Severo, N. Reis, L.C.G. Bazana, D.F. Dalla Lana, T. Vieceli, C.V.W. Riche, F.M. Perez); Santa Casa de Porto Alegre, Porto Alegre (A.C. Pasqualotto, C.V.W. Riche); University of Manchester, Manchester, UK (D.W. Denning); Duke University, Durham, North Carolina, USA (T. Le, I. Schwartz); University of Witwatersbrand, Johannesburg, South Africa (N.P. Govender); Westerdijk Fungal Biodiversity Institute, Utrecht, the Netherlands (F. Hagen); Fundação Oswaldo Cruz, Rio de Janeiro, Brazil (R.M. Zancope-Oliveira); IMMY, Norman, Oklahoma, USA (D.H. Caceres, T. Chiller); Université de Guyane, Cayenne, French Guiana (U. Francoise, M. Nacher, A. Adenis); Fluminense Federal University, Niteroi, Brazil (A. Guimaraes); Federal University of Ceara, Fortaleza, Brazil (L.S. Damasceno, T.M.J. Leitao); Pontifical Catholic University of Rio Grande do Sul, Porto Alegre (D.R. Falci); Hospital de Clínicas de Porto Alegre, Porto Alegre (D.R. Falci); Universidad del Rosario, Bogotá, Colombia (B.L. Gomez); Emilio Ribas Institute, São Paulo, Brazil (J.E. Vidal); Hospital Geral de Roraima, Boa Vista, Brazil (L.E. Galan); Federal University of Rio Grande, Rio Grande, Brazil (M.O. Xavier); Universidad Nacional del Litoral, Santa Fe, Argentina (G.G. Effron); University of Exeter, Exeter, UK (G. Brown); MiraVista Diagnostics, Indianapolis, Indiana, USA (N. Barros); Indiana University, Indianapolis (N. Barros); Pontifical Catholic University of Goias, Goiania, Brazil (C.M. Godoy, T. Fraga, R.B.A. Soares); Federal University of Santa Maria, Santa Maria, Brazil (A.V. Schwarzbold); Hospital General de Medellín, Medellín, Colombia (I. Berrio); Hospital Nossa Senhora da Conceição, Porto Alegre (M.G. de Melo); Hospital Vila Nova, Porto Alegre (N. Reis); Brasilia University, Brasília, Brazil (B.G. Tenorio, M. de M. Teixeira); Bahia State University, Salvador, Brazil (C.J. de C. Bastos); Federal University of Rio Grande do Norte, Natal, Brazil (M.B. Bay); Tropical Medicina Foundation, Manaus, Brazil (M.V.G. de Lacerda); Hospital General San Juan de Dios, Guatemala City, Guatemala (E. Arathoon); National Laboratory of Clinical Mycology, Buenos Aires, Argentina (C. Canteros); University of Minnesota, Minneapolis, Minnesota, USA (A.C. Pasqualotto, D. Boulware, N.C. Bahr); Spanish National Centre for Microbiology, Madrid, Spain (A. Alastruey-Izquierdo); Global Action for Fungal Infections, Geneva, Switzerland (A. Alastruey-Izquierdo, T. Chiller, J.L.R. Tudela); University of Lagos, Lagos, Nigeria (R. Oladele); Pan American Health Organization, Washington, DC, USA (F.M. Perez)

**Keywords:** histoplasmosis, mycoses, fungi, Histoplasma capsulatum, respiratory infections, International Society for Human and Animal Mycology, ISHAM

## Abstract

Histoplasmosis remains a neglected yet deadly fungal infection, disproportionately affecting persons living with HIV/AIDS and other immunocompromised populations in endemic regions. Despite the World Health Organization’s designation of *Histoplasma* as a high-priority pathogen, the disease remains underdiagnosed and excluded from national surveillance systems, resulting in delayed treatment and high death rates. To coordinate a global response, the International Society for Human and Animal Mycology convened a Histoplasmosis Working Group during its 2025 congress in Brazil. Experts engaged in structured discussions across 5 domains: awareness, research, diagnostics and treatment, capacity building, and fungal biology. The group highlighted persistent diagnostic delays, underuse of antigen testing, and poor access to liposomal amphotericin B and itraconazole. Innovations such as lateral flow assays and molecular tools were discussed, alongside the need for biobanks and validated diagnostic algorithms. A global 90–90–90 target for histoplasmosis by 2030 was proposed to improve diagnosis, treatment, and survival.

Histoplasmosis, caused by thermally dimorphic fungi of the genus *Histoplasma*, remains a neglected and life-threatening mycosis that disproportionately affects immunocompromised populations, particularly persons living with HIV/AIDS (PLHA) (*1*). Although *Histoplasma* has been identified as a high-priority fungal pathogen by the World Health Organization (WHO) at a global scale (*2*,*3*), histoplasmosis is not included in national epidemiologic surveillance systems in most endemic countries. As a result, its actual prevalence remains largely unknown, leading to widespread underdiagnosis and delays in treatment. Those challenges contribute to a high case-fatality rate, especially in settings with limited access to non–culture-based diagnostic tools and essential antifungal medications.

Since 2013, a series of initiatives and targets have been launched to combat histoplasmosis, starting in the Americas and gradually expanding to a global scale. At that time, histoplasmosis was recognized among experts as a neglected disease, a lethal blind spot among international health organizations, responsible for a substantial proportion of AIDS-related deaths in Latin America and the Caribbean (LAC) (*4*,*5*). Two major expert meetings led to the establishment of regional goals aimed at improving access to diagnostic tools and antifungal treatments for histoplasmosis in LAC. The first, held in 2015, launched the 80 by 2020 initiative, which sought to expand diagnostic and antifungal treatment coverage by 80%. The second, in 2019, set a more ambitious target: 100 by 2025 (*6*,*7*). The 2019 meeting, supported by the Pan American Health Organization, brought together a diverse group of stakeholders, including nongovernmental organizations, industry representatives, and academy experts, and resulted in the Manaus Declaration and publication of the first international guidelines for managing histoplasmosis in PLHA (*8*). To consolidate previous efforts, additional meetings with histoplasmosis experts were held in Brazil to sustain momentum and address persisting gaps in diagnostic and treatment access (*9*). As an indicator of the effect of those initiatives across LAC, where most of the efforts were historically concentrated, repeated cross-sectional surveys led in 2018 (*10*) and 2025 (A.C. Pasqualotto et al., unpub. data) informed participants about the increase in *Histoplasma* antigen detection access (set at ≈40% with a notable >100% increase in the number of centers equipped in Brazil) and in liposomal amphotericin B availability (set at ≈60% with a notable 20% increase in Brazil). Building on those initiatives, in 2024, the International Society for Human and Animal Mycoses (ISHAM) created a dedicated Histoplasmosis Working Group to unite international experts and networks to advance a coordinated global response targeting the structural drivers, systemic gaps, and ongoing challenges of the disease worldwide.

The group held its inaugural meeting during the ISHAM 2025 Congress, which took place in Iguazu Falls, Brazil, during May 20–24, 2025. This gathering provided a critical platform for scientists, clinicians, public health officials, and advocates to share recent advances and address ongoing challenges in histoplasmosis research and response. This article summarizes the key themes presented during the meeting, organized into major thematic areas, and concludes with a proposal for a global 90–90–90 target for symptomatic histoplasmosis control in all populations-at-risk worldwide (Figure), modeled after the successful Joint United Nations Programme on HIV and AIDS (UNAIDS) strategy for the fight against HIV/AIDS (*11*).

**Figure:**
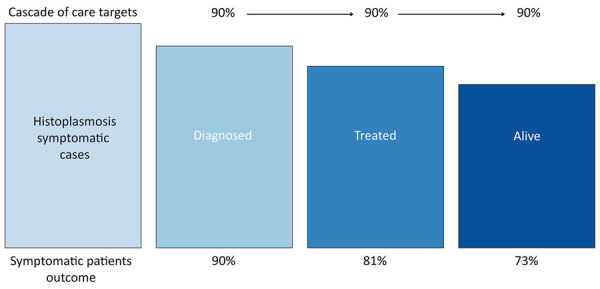
Proposed 90–90–90 global targets for symptomatic histoplasmosis control in all at-risk populations by 2030 from the International Society for Human and Animal Mycology 2025 Histoplasmosis Working Group.

Although disseminated histoplasmosis in advanced HIV disease (AHD) remains the top diagnostic and treatment priority because of substantial global prevalence and high case-fatality rates, other forms of histoplasmosis also greatly contribute to the case prevalence, major illness, and, in some cases, death. Examples include adrenal histoplasmosis (mostly occurring in immunocompetent patients), which can be mistaken for adrenal tuberculosis (TB) and may manifest with adrenal crisis (*12*), especially if treated with empirical rifampin. Central nervous system histoplasmosis, a condition that may mimic tuberculous or cryptococcal meningitis, is most often documented in immunocompromised patients but can also occur in previously healthy persons (*13*). Chronic pulmonary histoplasmosis can be mistaken for TB and has recently been reported to frequently affect persons with multidrug-resistant TB (as seen in Indonesia) and those with severe pulmonary disease (*14*). Gastrointestinal histoplasmosis can be mistaken for inflammatory bowel disease and can have potential dire consequences if treated with corticosteroids (*15*). In addition, other immunocompromised patients, including those in nonendemic countries, can have onset of disseminated histoplasmosis after reactivation of latent infection. However, in light of the substantial burden of histoplasmosis among immunocompromised persons, this article places primary emphasis on that population.

## ⁠Raising Awareness through Advocacy and Education

The ISHAM Histoplasmosis Working Group meeting opened with the acknowledgment that histoplasmosis remains largely invisible to many health systems and funding agencies. Public health campaigns and clinical awareness programs are scarce, even in high-prevalence countries. Histoplasmosis often is misdiagnosed as other diseases, particularly TB, and particularly in persons with AHD, leading to missed diagnoses and fatal delays in treatment (*16*,*17*). Travel and climate change may contribute to the emergence of histoplasmosis in nonendemic regions, resulting in new areas at risk (e.g., having soils suitable for *Histoplasma* growth) and relevant numbers of clinical cases newly reported, potentially increasing the disease prevalence in areas previously considered unaffected (*18*–*20*).

Meeting participants emphasized the urgent need to improve medical education, beginning with medical and school-level education and continuing through professional career development. Medical curricula and clinical training must include endemic fungal infections and have content tailored to local and epidemiologic relevance. Enhancing awareness among healthcare professionals is a critical step toward achieving earlier diagnosis and better outcomes in patients with histoplasmosis (*21*). The availability of diagnostic tests and antifungal drugs alone does not guarantee clinical impact. Without proper training, standardized protocols, and clinical awareness, diagnostic tools and antifungal therapies may remain underused or misapplied, ultimately limiting their effect on patient outcomes (*22*). Educational activities also should target decision-makers within national health systems, raising awareness about the importance of histoplasmosis, following the example of the development of WHO recommendations on the management of HIV-associated histoplasmosis and the ongoing WHO initiatives on the fungal pathogen priority list.

Although histoplasmosis remains a major health concern among PLHA, it also poses a considerable threat to other immunocompromised populations, including solid organ transplant recipients, patients with hematologic malignancies, persons receiving biologic or immunotherapy medications, and persons with autoimmune diseases. Furthermore, immunocompetent persons residing in endemic regions are not exempt from risk. Despite this wide range of vulnerable populations, histoplasmosis frequently is excluded from differential diagnosis algorithms and remains underrecognized in many clinical settings.

Advocacy has been identified as a critical strategy to mobilize political support, highlighting the urgent need to include histoplasmosis in national lists of notifiable diseases and to systematically integrate fungal infections into HIV, TB, and neglected tropical disease control programs. To improve early diagnosis and outcomes, biomarker standardization across different risk groups is essential, given that diagnostic data derived from cohorts of PLHA cannot be reliably extrapolated to other populations. Expanding awareness and diagnostic strategies to encompass all at-risk groups therefore is vital. Finally, members strongly encouraged intersectoral collaboration, involving ministries of health, academia, nongovernment organizations, and international agencies, to strengthen surveillance, diagnosis, and treatment capacity for histoplasmosis on a global scale. Engaging industry and further developing public–private partnerships also was recognized as a key component of an advocacy strategy.

## ⁠Advancing Collaborative Research

In recent years, interest in fungal diseases, including histoplasmosis, has increased. For the first time in decades, some clinical studies, laboratory evaluations, and implementation science projects are underway. During the meeting, members strongly emphasized the importance on leveraging existing biobanks, data, and specimens from multicenter clinical trials (including those not specifically aimed at histoplasmosis) and shared databases to accelerate the development of diagnostic tools, treatment algorithms, and predictive models. The recent development of point-of-care lateral flow assay tests has opened questions about whether a role exists for screening asymptomatic persons at high risk for disseminated disease (e.g., AHD) (*23*–*27*). Given the limited published data and conflicting information on the clinical effect of screening through circulating antigen detection in asymptomatic persons (*28*,*29*), the group proposed conducting prospective clinical studies to evaluate the utility of antigen-based screening in patients with AHD and in other at-risk populations.

Moreover, emphasizing that *Histoplasma* is a primary fungal pathogen, not merely an opportunist pathogen, is important. Although *Histoplasma* infection may not typically cause severe disease in immunocompetent persons, it can still lead to clinically relevant illness, notably within the context of outbreaks of various magnitude and outcomes (*30*,*31*). As such, strategies for detection and treatment should not be limited to patients with profound immunosuppression but should be extended to immunocompetent persons among residents of endemic area, travelers, and ecotourists. Moreover, oligosymptomatic or asymptomatic cases also occur in persons with mild immunosuppression, who could benefit from early diagnosis and treatment, leading to favorable outcomes.

Another key topic of discussion is the external validation of clinical scoring systems and diagnostic algorithms, particularly in settings where fungal culture and histopathologic analysis remain unavailable. Cross-border research collaborations and regional networks are proving vital in this effort. Strengthening laboratory and research capacity, especially in low- and middle-income countries, will be critical to ensuring sustainability and scientific equity. Establishing a broad, well-coordinated network capable of generating high-quality data are essential for refining algorithms and scoring systems. Such a platform also would support the integration of machine learning and artificial intelligence tools to enhance our understanding of *Histoplasma* behavior across a range of high-risk conditions. Previous studies using large language models showed the critical improvements in helping clinicians consider histoplasmosis and teaching physicians on how to prompt artificial intelligence (*32*,*33*). The study group is well positioned to play a pivotal role in driving such initiatives forward.

## ⁠Building Capacity

Diagnostic and therapeutic capacity for histoplasmosis remains grossly inadequate in many settings, even in highly endemic areas. For example, no country in Africa offers widespread testing for *Histoplasma* antigen across its healthcare services (*34*), and much of LAC and Southeast Asia also have limited access (*35*,*36*). Participants shared various capacity-building initiatives efforts, including regional training programs, laboratory mentorship schemes, and introduction of point-of-care antigen testing in clinics. Several successful models demonstrated the feasibility of establishing fungal diagnostic platforms, even in low-resource settings.

Moreover, a persistent cognitive bias seems to exist among clinicians: a tendency to default to a diagnosis of TB in HIV-associated febrile or pulmonary illness (*37*). Overcoming this diagnostic inertia is critical to improving patient outcomes. One participant summarized, “When tuberculosis is ruled out, histoplasmosis should be considered as a relevant differential diagnosis; moreover, even when tuberculosis is confirmed, histoplasmosis may still coexist and must not be overlooked.”

## ⁠Expanding Access to Modern Diagnostics and Treatment

Participants emphasized the urgent need to expand access to rapid and sensitive diagnostic tools for histoplasmosis. Antigen detection tests, particularly those using lateral flow devices, were repeatedly described as transformative, providing same-day results rather than the many days or weeks required by conventional culture or histopathologic methods. This rapid turnaround enables timely initiation of therapy, which is critical for improving outcomes (*8*,*25*,*38*). Despite their proven utility, those diagnostic tools remain largely underused because of limited manufacturing sources, regulatory barriers, and limited availability. However, participants noted that the primary constraint may not lie in the availability of the tests themselves but rather in inefficiencies of procurement processes and suboptimal integration of diagnostic services into health systems. Quantitative antigen testing may serve not only as a prognostic biomarker but also as a potential tool for monitoring treatment response. This point is especially relevant in the context of drug-drug interactions associated with itraconazole, which often are observed in persons with TB co-infection and in transplant recipients. Molecular platforms, including PCR and loop-mediated isothermal amplification–based assays, also were discussed as promising innovations (*39*), particularly for diagnosing cases with atypical manifestations or low fungal prevalence, but those platforms also will have similar limitations and still need to be developed.

At the same time, access to critical antifungal therapies remains inconsistent. The distribution and use of liposomal amphotericin B and itraconazole, essential treatments for severe and disseminated histoplasmosis, are hampered by regulatory bottlenecks, economic constraints, and logistical barriers (*10*). Participants strongly advocated for the inclusion of these medications on national essential medicines lists and in procurement strategies aligned with existing HIV and tuberculosis programs. Investment in secure supply chains, rational prescribing practices, and postmarket surveillance also were recognized as essential to ensuring long-term sustainability.

Concerns remain about the affordability and accessibility to liposomal amphotericin B, especially in low- and middle-income countries (*40*). Although generic formulations are increasingly available, substantial structural challenges related to procurement processes, supply chain management, and financing mechanisms have yet to be addressed, and concerns regarding their clinical efficacy may also exist. Participants emphasized the potential benefits of simplified and standardized treatment protocols, particularly if they are integrated into HIV clinical pathways, to improve outcomes and reduce delays (*41*). Ensuring the availability of newer antifungal drugs with better long-term safety profiles and less potential for drug interactions is essential because of adverse effects associated with long-term itraconazole use and major drug interactions between itraconazole and rifampin in patients with concurrent TB and drug interactions between itraconazole and chemotherapeutic agents metabolized through the common hepatic cytochrome P450 system in cancer patients. Furthermore, highlighting the poor oral bioavailability and unpredictable pharmacokinetics of itraconazole capsules is important and emphasizes the need for broader access to formulations with improved bioavailability.

Establishing important public–private partnership models and engaging with industry are key to bringing the diagnostics and treatment to the patients. Further work to build these connections and solutions is needed. Early investment from foundations and government will be key to helping develop antifungals.

## ⁠Understanding Pathogen Diversity and Host Interactions

A deeper understanding of *Histoplasma* strain diversity and host–pathogen interactions is essential for refining diagnostic and guiding therapeutic strategies. Recent advances in molecular epidemiology have reviewed the existence of distinct cryptic species and phylogenetic clades within the genus *Histoplasma*, some of which may exhibit different clinical manifestations or antifungal susceptibilities, findings that have important implications for public health interventions (*42*–*44*). However, the clinical and epidemiologic implications of that diversity remain mostly underexplored, hindering optimal case management and undermining surveillance efforts. The working group emphasized the urgent need to expand genomic surveillance of *Histoplasma* strains across endemic regions, coupled with standardized clinical and phenotypic characterization.

Currently, only unapproved skin tests are available to determine latent infection, increasing the risk for reactivation of *Histoplasma*, which highlights a considerable gap in diagnostic tools to determine disease exposure and risk for disease reactivation in susceptible persons undergoing impending immunosuppressive therapy. Although preliminary data suggest the potential utility of interferon-γ release assays for histoplasmosis, such approaches remain investigational and require further validation (*45*).

Research into the host immune response, including innate pathways, T-cell responses, cytokine profiles, vaccines, and passive immunization strategies, is critical to elucidate host immune susceptibility and host immune contribution to treatment outcomes, particularly in immunocompromised populations (*46*,*47*). Efforts to identify potential biomarkers of disease severity also was discussed as a priority area. In addition, animal histoplasmosis may serve as indicator of environmental exposure and emerging hotspots. Cross-disciplinary collaboration with veterinarians, environmental biologists, and epidemiologists was proposed as a strategic next step to enhance early detection and surveillance. Early detection of histoplasmosis is essential to prevent progression to disseminated disease.

One important question to address is: why do not all exposed and at-risk persons experience onset of the disease? Beyond host–pathogen interactions, a need to better understand individual risk factors persists, requiring deeper investigation into whether specific host characteristics influence the acquisition, clinical progression, and diverse manifestations of histoplasmosis (*48*). Enhanced risk assessment could ultimately lead to more targeted prevention and early treatment strategies.

## ⁠A Roadmap for the Future: 90–90–90 Targets for Histoplasmosis

Taking advantage of previous initiatives and targets, and guided by the overarching goal of reducing histoplasmosis-related deaths, participants decided to galvanize the global response and committed to a structured 90–90–90 target for histoplasmosis control by 2030 (Figure), in which:

- 90% of patients have their illness diagnosed, ensuring that most symptomatic and high-risk histoplasmosis cases are identified early through improved clinical awareness and expanded access to antigen-based and, ideally, point-of-care diagnostics.
- 90% of diagnosed patients are treated according to guidelines, guaranteeing the timely initiation of effective antifungal therapy, based on protocols tailored to disease severity, immune status, and available resources.
- 90% survival of patients who have their illness diagnosed and treated, ensuring that the ultimate goal of any action, whether through bottom-up or top-down initiatives, is to reduce suffering and save lives, especially in settings where histoplasmosis continues to cause substantial illness and death rates (≈30%).

Achieving this global vision will require unprecedented collaboration among national health systems, international agencies, research institutions, and funding bodies. Equally important is the need to monitor progress through improved surveillance systems and robust outcome indicators, which are essential for informing programmatic adjustments and measuring effect. In that context, developing automated surveillance systems powered by artificial intelligence should be strongly advocated. Such tools are critical to alleviating the burden of manual data entry into multiple databases, systems that often rely on limited variables and lack the granularity necessary for meaningful analysis. Developing such systems becomes particularly urgent in resource-limited settings, where healthcare workers are already overstretched and lack the time or capacity to collect and interpret high-quality data. Automation offers a pathway to substantially enhance the efficiency and accuracy of data collection, ultimately strengthening health system responsiveness and decision-making.

## Monitoring Progress

Progress in addressing histoplasmosis can be monitored by using a combination of programmatic indicators, surveillance platforms, and implementation research embedded in existing AHD frameworks. Over the past decade, Global Action for Fungal Infections (GAFFI) and partner institutions have demonstrated that real-world packages of care integrating *Histoplasma* antigen testing, standardized treatment protocols, and access to essential antifungal drugs can be implemented within routine HIV services. Those initiatives generate measurable indicators, including diagnostic coverage, treatment uptake, and survival, which provide empirical baselines against which progress can be tracked over time. Harmonization of case definitions, diagnostic algorithms, and outcome measures across GAFFI-supported projects and academic networks enables aggregation of data and comparison across settings, transforming previously fragmented efforts into a coordinated monitoring system.

At a regional level, the Pan American Health Organization monitors AHD through a structured framework and an interactive regional dashboard that consolidates data from multiple sources, including UNAIDS Global AIDS Monitoring (https://www.unaids.org/en/global-aids-monitoring), UNAIDS Spectrum estimates (https://www.unaids.org/en/dataanalysis/knowyourresponse/HIVdata_estimates), TB reports, and direct country reporting. This platform enables dynamic tracking of AHD prevalence, opportunistic infections (including histoplasmosis), TB-HIV co-infection, and deaths at national and regional levels. By integrating histoplasmosis-relevant indicators into an existing, routinely updated AHD surveillance architecture, countries can monitor trends, identify diagnostic and treatment gaps, and adjust programs in near real-time without creating parallel reporting systems.

Together, such mechanisms provide a pragmatic and scalable approach to monitoring progress in histoplasmosis control. Embedding histoplasmosis indicators within established AHD surveillance platforms, while leveraging GAFFI- and academic-led implementation networks, enables continuous assessment of diagnostic coverage, guideline-concordant treatment, and patient outcomes. This combined strategy is well suited to support measurable targets, such as a 90–90–90 framework for histoplasmosis, while remaining flexible enough to incorporate new diagnostics, treatments, and policy priorities as the field evolves.

## Conclusion

The ISHAM Histoplasmosis Working Group reaffirmed that histoplasmosis is both a treatable disease and a public health emergency in many parts of the world. Tools to diagnose and treat now exist but remain inaccessible to many persons who need them most, and death rates remains unacceptably high. A call for action grounded in the 90–90–90 framework offers a practical and measurable roadmap for change. Together with political will, strengthened scientific collaborations, and targeted resource mobilization, the global community can reduce the toll of this neglected mycosis and fulfill the broader goal of equitable access to infectious disease care across all settings.
